# The British Journals, &c.: Surgery

**Published:** 1842-04

**Authors:** 


### SURGERY.

Prolapsus of the Uterus and anterior Wall of the Bladder. A servant, aged eighteen, while violently exerting herself "heard her back crack," and immediately after felt a bearing-down sensation in the pelvis, and a dragging sensation in the loins. These symptoms, along with difficulty in voiding urine and feces, continued for ten days, when a small tumour, which gradually increased to the size of an orange, protruded from the vagina. Non-retention of urine and other consequences followed, which impaired the girl's health, and all means proving fruitless, it was resolved to perform the operation of episoraphie, as recommended by Dr. Fricke of Hamburgh.

Sept. 2. The bowels having been previously evacuated, and the hair removed from the parts, the patient was placed on a table, in the same position as for lithotomy, without, however, tying the hands and feet. The thighs being well separated by two assistants, I took hold of the left labium and transfixed it obliquely about the middle with a narrow bistoury three-quarters of an inch from the edge, and in such a manner as to include more of the skin than of the mucous membrane; the knife was then carried rapidly downwards in the same direction to the raphe, half an inch or so in front of the anus; the superior attachment of this flap was next divided, by carrying the incision upwards as high as on a level with the meatus urinarius. The same was repeated on the opposite side; after which the frenulum, and other parts included within the angle formed by the union of the two incisions in front of the anus, were carefully dissected off. The two surfaces thus formed extended from opposite the urethra to within half an inch of the anus, each being about two inches long, and varying in breadth from an inch posteriorly to half an inch anteriorly. The hemorrhage was so trifling, as merely to require the torsion of one small vessel: the oozing having ceased, six strong hempen sutures were passed through the entire thickness of the denuded surfaces, and tied moderately firm. The first one was applied a few lines in front of the anus, and the last one immediately below the meatus urinarius.

The operation was performed in September, appropriate treatment was adopted, and by Nov. 20, the cure was complete.—*Lancet*, No. x. *Dec.* 4, 1841.

---

WOUND OF THE AXILLARY ARTERY. The only point worthy of notice in this case (which terminated fatally), is its having furnished the reporter an opportunity of observing that, in the operation for tying the axillary artery below the clavicle, the axillary vein is not always, as is usually described, distended with blood, as to oppose an obstacle to the taking up of the artery; nor is the vein always easily recognizable, as is also alleged, by its peculiar colour. In the present case the vein was collapsed, and could not be distinguished either by protuberance or colour, from the fascia covering the vessels. The pulsation of the artery was felt distinctly through the vein; and the latter, had great care not been taken in detecting and avoiding it, might readily have been confounded with the fascia, and included in the incisions.—Dr. SCRATCHLEY, *Provincial Medical and Surgical Journal*, No. ix. *Nov.* 27, 1841.

---

SUCCESSFUL OPERATION FOR PROLAPSUS UTERI. The bladder being previously emptied, the patient was laid over a bed upon her face, and three large folds were excised from the posterior and lateral parts of the vagina. There was profuse hemorrhage, and great difficulty was experienced in securing the vessels. Ligatures kept the parts together, and there was ultimately a complete cure.—Mr. MORRIS, *Medical Gazette*, No. xiv. *Dec.* 24, 1841.

---

DISSECTION OF A CASE OF SPINAL CURVATURE. The object of this paper is to discountenance the operation of dividing the muscles in cases of spinal curvature. Even division of the sacro-lumbar fascia seems to be of little use in restoring the spinal column to the recto-linear state, and in the present case, an inspection of the bodies of the vertebræ and of the intervertebral substance, both of which had greatly lost volume on the concave side of the spine, made it evident that the obstacle to the bringing back of the column to the straight position, was what the author calls "organic," in other words, mechanical and structural; and such as the freest section both of the fascia and muscles could have had no effect in recovering.—Mr. GAY, *Medical Gazette*, No. xiv. *Dec.* 24, 1841.

---

NEW TREATMENT OF ANEURISM. The author, who has not yet had an opportunity of reducing his theory to practice, proposes that a needle be introduced into the aneurismal sac, and the coagulum broken up; the fragments of which becoming impacted in the discharging orifice of the sac, and the passage of blood being thereby arrested, there could be no doubt "but that the cavity would soon become obliterated."—Mr. BLAKE, *Medical Gazette*, No. xvi. *Jan.* 7, 1842.

COMPARATIVE MERITS OF AMPUTATION IMMEDIATELY BELOW THE TUBEROSITY OF THE TIBIA, OR AT OR BELOW THE MIDDLE OF THE LEG. The points insisted on in this able paper are, that we ought never to remove more of the leg than will ensure that the parts which form the stump are sound; on the principle that, *cæteris paribus*, the nearer the truuk, the more dangerous the operation: that, if provided with a suitable apparatus, namely, the pin which admits of walking being performed with the knee straight, the patient can make a much freer use of his stump in all positions, except the erect and recumbent, than if the operation had been performed immediately below the tuberosity of the tibia. The author cites the case of a sailor, on whom amputation of the lower part of the leg had been performed; and who, having had a hook attached to the forepart of his short pin, climbed the rigging nearly as nimbly as before he lost his leg.—Dr. ADAIR LAURIE, *Medical Gazette*, No. xviii. *Jan.* 21, 1842.

---

SUCCESSFUL CASE OF TRACHEOTOMY The patient, a female of fifty-five years, had suffered for two years from difficulty of breathing and of swallowing, and from pain referred to the site of the larynx. No evidence of disease in the lungs, heart, or great vessels existed to account for the attacks of dyspnœa, which at length became so urgent as to threaten life. The operation was performed on the 6th of December, with immediate relief; a small silver canula was introduced the same evening, and some auxiliary treatment adopted. On the 12th she could breathe freely through the natural passage. The cure was complete.—Mr. EWEN, *Provincial Medical and Surgical Journal*, No. xiii. *Dec.* 25, 1841.

---

PARAPHYMOSIS CURED WITHOUT OPERATION. The incision of the stricture in paraphymosis is pretty generally practised by surgeons, except when this disease occurs in children, and in such the reduction is often effected by pressure. I have seen repeated incisions fail of relieving the stricture, and leave foul and intractable sores, which have been extremely tedious and difficult to heal. I have always succeeded in reducing a paraphymosis, either in an adult or child, however long standing, without having recourse to the knife, by the following method: I place the patient against a wall, and take care that he is steadily supported by an assistant on each side; a piece of linen cloth is then laid over the glans, and with my thumbs I knead the blood out of it, drawing the prepuce forwards at the same time with two fore-fingers of each hand. A steady perseverance in this plan never fails, and, although the operation is a painful one, the patient is amply rewarded by the rapidity of the cure, which requires nothing more than the application of a saturnine lotion for two or three days. J. TOOGOOD, Esq., *Provincial Medical and Surgical Journal*, No. xvi. *Jan.* 15, 1842.

---

LIGATURE OF THE CAROTIDS. In this paper two points are insisted on: first, that, should circumstances demand the operation, both carotids may be simultaneously tied, without risk of ill consequences; and, secondly, that, after ligature of one or both of these arteries, the organ apt to suffer in consequence of the disturbance of the circulation caused by the operation is the *lungs* and not the *brain*. This is proved by the numerous experiments of M. Robèrt, performed on animals; the untoward symptoms which followed the operation developing themselves in the chest, and not in the cranium. The same fact is further illustrated by cases of Messrs. Ferguson, Liston, Lizars, Sir Astley Cooper, &c., which the author refers to, in which dispnœa and cough succeeded the operation, but which do not seem to have been properly understood, or to have attracted due attention or to have been treated, as the author thinks they ought to have been, by bloodletting. He therefore advises that, in plethoric subjects, the operation should be preceded by venesection; and that the same measure should be repeated after the operation, if any pulmonary symptoms supervene. He would extend the same rule and the same cautions to cases of operation on other great arterial trunks, such as the subclavians, the iliacs, and the femorals; after ligature of which, congestions of the thoracic and abdominal viscera are apt to follow. The author thinks that when, after ligature of the carotid, cerebral symptoms do supervene, these are not due to the change in the circulation, but to some injury inflicted during the operation on the nerves or vein accompanying the artery, or to casualties attending the wound.—James Miller, Esq. *London and Edinburgh Monthly Journal of Medical Science,* No. i. *Jan.* 1841.

---

Closure of the Os Uteri. The case was that of a married woman, who had not menstruated since the birth of her last child, two years previously. On examination, the os uteri was found closed. It was punctured, and two pints of fluid gushed out, with immediate relief to the patient: two pints more came away soon after. It appeared, on enquiry, that, immediately after her last parturition, she had been seized with inflammation of the neck of the womb, and that the lochia had never appeared.—Mr. Pugh, *Maryland Medical and Surgical Journal,* No. i. *July,* 1841.

---

Strangulated Hernia treated with Opium. A man aged fifty, with a large inguinal hernia, was seized with symptoms of strangulation on the 16th of November. The taxis being employed without effect, the operation was proposed, which the man would not submit to. On the 19th another gentleman saw the case, who recommended the author to try large doses of opium, as, he said, Mr. Bransby Cooper had informed him of a surgeon in Somersetshire who for the last eighteen years had so treated similar cases. The author immediately gave four grains of opium, which relieved the pain and sickness, but produced no change in the tumour. In four hours he repeated the opium, and in five hours gave a third dose of four grains. On the 20th the patient was free from pain and sickness, though the hernia was in the same state. At length, however, the bowels operated, without any apparent alteration in the tumour; from which time he recovered, and the hernia gradually disappeared.

Another case, that of a woman aged seventy, is reported, which had an equally satisfactory result.—Mr. Cooper, *Medical Gazette,* No. xxii. *Feb.* 18, 1842.

---

Case of successful Operation for Lateral Curvature. The subject of this operation was a young lady aged nineteen, naturally of a good constitution, but rendered feeble and nervous by the deformity of the spine. Dr. Brown did not perform this operation in a manner precisely similar to that described by the European surgeons. He made the puncture on a line with the last dorsal vertebra, carrying the knife on its flat side between the integuments and muscles nearly to the spinous process of the same vertebra. He then turned it, and divided the longissimus dorsi transversely; again turning the instrument, he ran it down near to the spinous processes for the space of two inches, and then up along the course of the spine two inches, dividing the whole of the spinal attachment of the serratus posticus inferior, making a subcutaneous longitudinal incision of four inches, involving of course a division of the attachments of the latissimus dorsi at these points; all of which was done through one cutaneous puncture. There was no bleeding of any consequence—probably not more than a tea-spoonful. A small piece of adhesive plaster was applied over the puncture, which being secured by a compress and roller, the young lady walked to her bed. The deviation between the shoulder-blades, which previous to the operation was three inches, was reduced in four days to one inch and a quarter. Extension having been applied by means of the inclined plane used in the institution, the lumbar curve has entirely disappeared, and she has gained one inch and a half in height.—Dr. Browne, *Boston Medical and Surgical Journal,* No. xvi. *Nov.* 24, 1841.

---

Deformity from Rheumatism. Operation. The author of this paper found the patient, a young woman of 24 years, in the following condition: the elbow joints were completely anchylosed; the knee-joints were bent upon the thighs at an angle of 45 degrees, were somewhat enlarged, and admitted but of slight motion; the muscles of both legs were atrophied.

From the circumstance of there being still motion left in the knee-joints, and as the chief obstacle to the action of these appeared to consist in the contracted and rigid state of the flexor muscles, the author divided by subcutaneous section, first, the semi-membranosus and tendinosus in the left leg, and then the biceps. A similar operation was performed on the right limb. Forty-eight hours from the operation, the wounds were quite healed, and splints with the revolving screw were applied. Eighteen days from the time of the sections, the apparatuses were removed, and the legs were quite straight. Ultimately the patient acquired a perfect use of her limbs. The case is interesting on account of the length of time during which the contraction of the joints had continued, which amounted to six years.—Mr. SYMES, *Medical Gazette*, No. xxiii. *Feb.* 25, 1842.

---

PROPRIETY OF OPERATING IN CASES OF SPREADING GANGRENE. The author gives a case illustrative of the propriety of amputation in case of spreading gangrene of the extremities.

Charles Tuck, a farm servant, aged 24, received the contents of a common fowling-piece in the hand, on Friday, the 4th of February, which passed up the flexor muscles and through the integuments of the fore-arm about three inches above the wrist. A neighbouring surgeon saw him soon after the accident, and directed cold applications and rest. On the following Monday he was admitted into the Bridgwater Infirmary. There was a ragged wound at each opening, the limb was but little swollen, and the only unfavorable appearance was, that the nails looked rather dark coloured. There was very little constitutional disturbance. He was put to bed, a poultice applied over the whole limb, and the usual treatment directed. On the next day the aspect of the limb was the same; but as there was more swelling in the evening and some heat, leeches and fomentations were ordered. He slept perfectly well until five o'clock on the following morning, Wednesday, when he was awoke by pain, which rapidly increased and became very severe. At seven o'clock the limb was found to be gangrenous to the elbow, and before his consent to its removal could be obtained, and the necessary preparation made, it extended so high up, as barely to leave room to amputate close to the shoulder-joint. There was no line of demarcation, but I cannot, at this distance of time, recollect the precise point to which the crepitus extended, but I am inclined to believe it was quite up to the joint, as it was debated whether it would not be the safest plan to remove the bone from the socket. Very little blood was lost during the operation; the constitutional irritation subsided in a few hours; the patient became tranquil and soon recovered.

The case proved fortunate, and the author very justly observes, that "it has always appeared to him better to offer to the patient the chance which a doubtful operation affords, than to leave him to certain death."—Mr. TOOGOOD, *Provincial Medical and Surgical Journal*, No. xxii. *Feb.* 26, 1842.

---

HYPERTROPHY OF THE PAROTID GLAND. This gland, in a child of 9 months, had attained an enormous size, extending "from a line drawn from the external angle of the orbit two thirds down the neck; the lower part of it hanging detached and resting on the upper part of the chest. It extended backwards behind the mastoid process, and occupied two thirds of the cheek in front. The child died of convulsions, and on examination of the tumour, it appeared to possess a perfectly granular structure, and to be a case of simple hypertrophy. During life, several surgeons had supposed it to be a case of aneurism by anastomosis, and had advised ligature of the carotid—an operation which, as the author remarks, would probably have had no effect in checking the tumour.—Mr. DUKE, *Provincial Medical and Surgical Journal*, No. xxi. *Feb.* 19, 1842.

